# Ancestral Gene Organization in the Mitochondrial Genome of *Thyridosmylus langii* (McLachlan, 1870) (Neuroptera: Osmylidae) and Implications for Lacewing Evolution

**DOI:** 10.1371/journal.pone.0062943

**Published:** 2013-05-23

**Authors:** Jing Zhao, Hu Li, Shaun L. Winterton, Zhiqi Liu

**Affiliations:** 1 Department of Entomology, China Agricultural University, Beijing, China; 2 California State Collection of Arthropods, California Department of Food and Agriculture, Sacramento, California, United States of America; International Atomic Energy Agency, Austria

## Abstract

The first complete mitochondrial genome of the lacewing family Osmylidae (*Thyridosmylus langii* (McLachlan, 1870)) (Neuroptera) was sequenced in this study. The genome is a circular molecule of 16,221 bp containing the typical 37 genes but is arranged in the same order as that of the putative ancestor of hexapod and lacks translocation of *trnC* as shared by all previously sequenced neuropteran mtDNAs. This reveals that *trnC* translocation does not represent an organizational synapomorphy in the mitochondrion for the entire Neuroptera clade. Comparative analysis of neuropteran tRNA genes reveals a relatively slow and conserved evolution of the mitochondrion throughout the order. Secondary structure models of the ribosomal RNA genes of *T. langii* largely agree with those proposed for other insect orders. Nevertheless, domain I of *T. langii rrnL* is consisted of nine helices rather than eight helices which is typical for neuropteran *rrnL*. Protein-coding genes have typical mitochondrial start codons, with the exception of *COI*, which uses the TCG start codon also found in Ithonidae and Chrysopidae. Like other neuropteran insects, the control region is the most AT-rich region and comparatively simple, with little evidence of conserved blocks or long tandem repeats. Considering the issues of base-compositional and branch length heterogeneity, we used a range of phylogenetic approaches to recover neuropteridan relationships and explored the effect of method choice on recovery of monophyly of Neuropterida: ((Neuroptera + Megaloptera) + Raphidioptera). The monophyly of Neuroptera and the more basal position of Osmylidae were also recovered by different datasets and phylogenetic methods.

## Introduction

The study of mitochondrial (mt) genome has yielded significant insights into different scientific disciplines including animal health, comparative and evolutionary genomics, molecular evolution, phylogeography, phylogenetics and population genetics [1], [2]. The insect mt genome is a circular, double-stranded molecule of 14–19 kb in length that comprises a set of 37 genes for 22 tRNAs, 2 rRNAs and 13 proteins [3]. More than 270 insect mt genomes have been sequenced, representing 26 of the 30 orders, with most from the mega-diverse orders such as Diptera, Lepidoptera, Hemiptera, Coleoptera and Hymenoptera. Based on a series of analyses, mt genomes may be superior to individual nuclear gene or partial mt genes to reconstruct phylogenetic relationships as they possess a number of evolutionarily interesting and potentially informative features such as length variation, altered tRNA anticodons or secondary structures, atypical start codons, base compositional bias, codon usage, and gene rearrangement [4]–[6].

Neuropterida contains at least 6,500 described valid species divided into three orders: Raphidioptera (with slightly more than 200 species in two families), Megaloptera (with about 300 species in two families) and the extremely heterogeneous Neuroptera (with 6,000 species in 15 families) [7], [8]. Relationships within Neuropterida have been controversial over time. The traditional view is a sister group relationship between Megaloptera and Raphidioptera, which was corroborated by Wheeler et al. (2001) and Wiegmann et al. (2009) [9]–[11]. This view has been challenged recently with some researchers proposing that Raphidioptera are sister to Megaloptera + Neuroptera [8], [12], [13]. Megaloptera and Raphidioptera exhibit the typical insect ancestral gene order [14]–[16]. Conversely, Neuroptera with only six mt genomes fully sequenced to date, all share the common feature of translocation of *trnC* [15], [17]–[19].

Neuropteran family-level relationships have been controversial. The first quantitative cladistic analysis of neuropteran relationships based on adult and larval morphological characters divided Neuroptera into three suborders: Hemerobiiformia, Myrmeleontiformia and Nevrorthiformia [20]. The former two suborders were proposed as sister groups and comprise most neuropteran species, while Nevrorthiformia includes the single small family Nevrorthidae, supposedly representing the sister group to the rest of the order. The monophyly of the suborder Hemerobiiformia was not recovered with any satisfactory support when originally coined [20] and has been repeatedly shown to be paraphyletic in all subsequent quantitative analyses based on morphological or molecular data [8], [12], [13]. In a detailed analysis of Neuropterida using combined molecular and morphological data by Winterton et al. (2010), Hemerobiiformia were recovered as a paraphyletic grade containing Nevrorthiformia, with Myrmeleontiformia as a monophyletic derived clade [8].

Here, we present the first complete mt genome sequence of *Thyridosmylus langii* (McLachlan, 1870), a representative of Osmylidae. Osmylidae are considered one of the more basal groups of Neuroptera, exhibiting many plesiomorphic characteristics [8], [11], [12], [19]. Winterton et al. (2010) recovered Osmylidae as sister to Sisyridae and Nevrorthidae in a near-basal clade that originated during the Permian [8]. Characterization of the mt genome of Osmylidae provides an important opportunity to understand the sequence of evolution of the mitochondrion in Neuroptera, especially with reference to gene order, insertion and deletion events and ribosomal secondary structure. We provide analyses of the gene order, nucleotide composition, codon usage, compositional biases, RNA secondary structure, the assessment of base compositional and branch length heterogeneity and evaluate the phylogenetic relationships among the three orders of Neuropterida based on a range of phylogenetic approaches. This work reveals that the *trnC* translocation, which is absent in *Thyridosmylus*, occurred within higher Neuroptera and cannot be used as a synapomorphy characteristic for all Neuroptera.

## Results and Discussion

### Genome organization and structure

**Figure 1:**
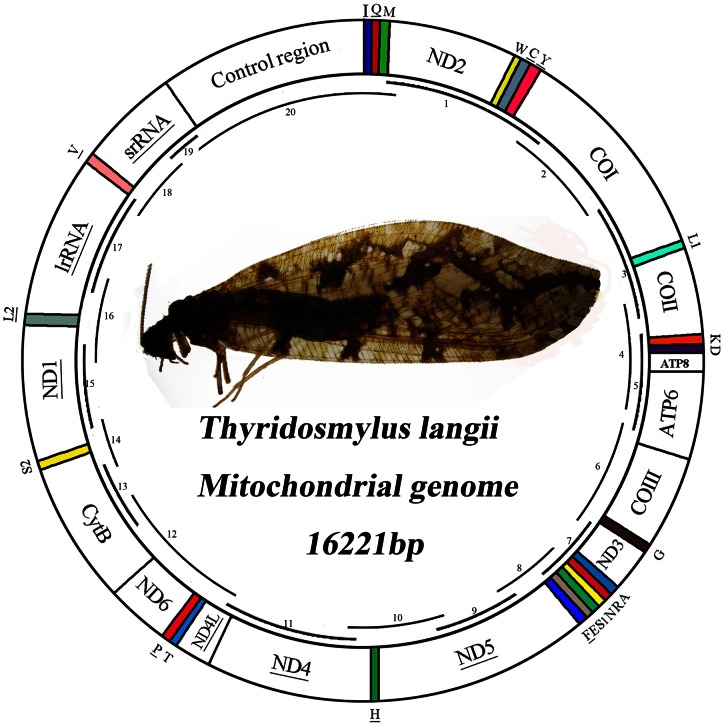
Map of the mt genome of *T. langii*. Transfer RNAs are denoted by the color blocks and are labeled according to the IUPACIUB single-letter amino acid codes. Gene name not underlined indicates the direction of transcription from left to right, and with underline indicates right to left. Overlapping lines within the circle denote PCR fragments amplified used for cloning and sequencing.

The mt genome of *T. langii* is a double-stranded circular DNA molecule of 16,221 bp in size and has been deposited in the Genbank under accession number (KC515397). This mt genome contains the entire set of 13 protein-coding genes (PCGs), 22 tRNA genes, two rRNA genes, and a control region (also called A+T-rich region) that are typically present in metazoan mt genomes (Fig. 1). The arrangement of mt genes of *T. langii* is the same as the putative ancestor of insects [21]–[26] and differs with the other six previously sequenced neuropteran mt genomes which share a common synapomorphic feature of transposition of *tRNA^Trp^* and *tRNA^Cys^* genes. The rearrangement of *tRNA^Cys^*–*tRNA^Trp^*–*tRNA^Tyr^* (CWY) was considered as a potential synapomorphic character for Neuroptera or some subgroup within the order in the previous study [15]. This opinion is further corroborated in our study that the rearrangement of CWY is just synapomorphic for some subgroup within Neuroptera. The likely explanation for transposition of adjacent genes in Neuroptera involves tandem duplication of the entire region affected by the transposition followed by inactivation and loss of the first copy of the second gene [21].

Genes are encoded by both strands of *T. langii* mt genome: 23 genes on the majority strand (J-strand) with the rest on the minority strand (N-strand). Eleven pairs of adjacent genes in the mt genome of *T. langii* overlapped by 1–8 bp and involved a total of 38 bp which make the genome relatively compact (Table S1). Two pairs of overlapping genes are common to all sequenced mt genomes of lacewings: *ATP8*/*ATP6* and *ND4*/*ND4L*, and share a near identical seven bp sequence (ATGNTAA), with the exception of the ascalaphids, in which this consensus-overlapped sequence were not found in the *ND4*/*ND4L* gene pair.

### Transfer RNAs

**Figure 2:**
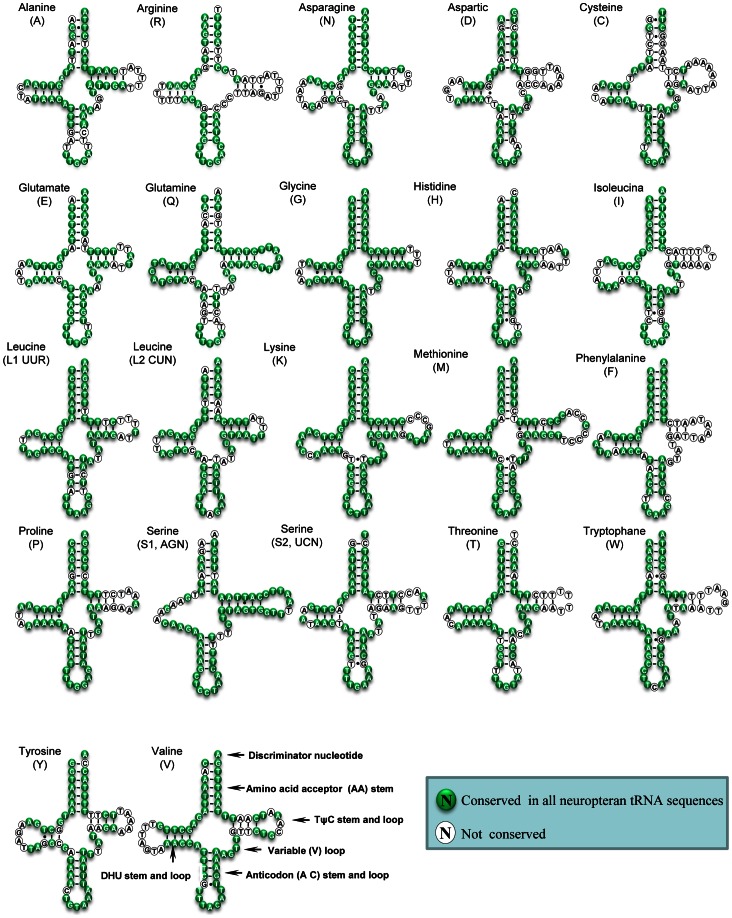
Inferred secondary structure of 22 tRNAs of *T. langii* mt genome. 22 tRNAs are labeled with the abbreviations of their corresponding amino acids. Dashes (−) indicate Watson-Crick base pairing and dots (•) indicate unmatched base pairing.

The mt genome of *T. langii* consists of all 22 tRNA genes, ranging from 64 to 73 bp. Most of them were detected using tRNAscane-SE [27] with two exceptions of the *tRNA^Arg^* and *tRNA^Ser(AGN)^* genes. Sequences of these two tRNAs were determined through comparison with previously published neuropteran mt genomes [14], [15], [18], [19]. All tRNAs could fold into the typical cloverleaf structure except for *tRNA^Ser(AGN)^*, in which its dihydrouridine (DHU) arm simply formed a loop (Fig. 2).

Based on the secondary structure, a total of 23 mismatched base pairs are found in the *T. langii* tRNAs: eighteen of them are G–T pairs, which form a weak bond, located in the AA stem (3 bp), T stem (3 bp), AC stem (5 bp) and the DHU stem (7 bp); the remaining five pairs include T–T mismatches (3 bp) in the AA stem of *tRNA^Leu^*, the T stem of *tRNA^Trp^* and the AC stem of *tRNA^Leu^*, respectively; C–T (1 bp) mismatches in the AC stem of *tRNA^Met^*; A–G (1 bp) mismatches in the AA stem of *tRNA^Trp^*. Mismatches observed in tRNAs are common in arthropod mt genomes and may be corrected through RNA editing processes [28].

Comparative analyses of secondary structures of neuropteridan tRNAs were undertaken based on generating a multiple alignment of tRNAs across exemplars and the percent of identical nucleotides (%INUC) (i.e. conservation) calculated. All tRNAs exhibited high levels of conversation with the average 89%INUC. The pattern of nucleotide conservation was obviously J strand-biased. Indeed, *trnG* and *trnK*, which showed the highest levels of nucleotide conservation (%INUC ≥95%), were all located on the J strand; while *trnR* and *trnC* showed the lowest levels of nucleotide conservation (%INUC≈80%) on both strands. Among neuropteran tRNAs, nucleotides at the AA and AC stems and loops were conserved: *trnG* and *trnN* showed 100%INUC in these two arms; and variations were largely restricted to the TΨC, DHU, and extra loops (Fig. 2). The high levels of nucleotide conservation characterize the low rate of nucleotide substitution and reveal that Neuroptera has in general experienced a slow and conservative evolution.

### Ribosomal RNAs

**Figure 3:**
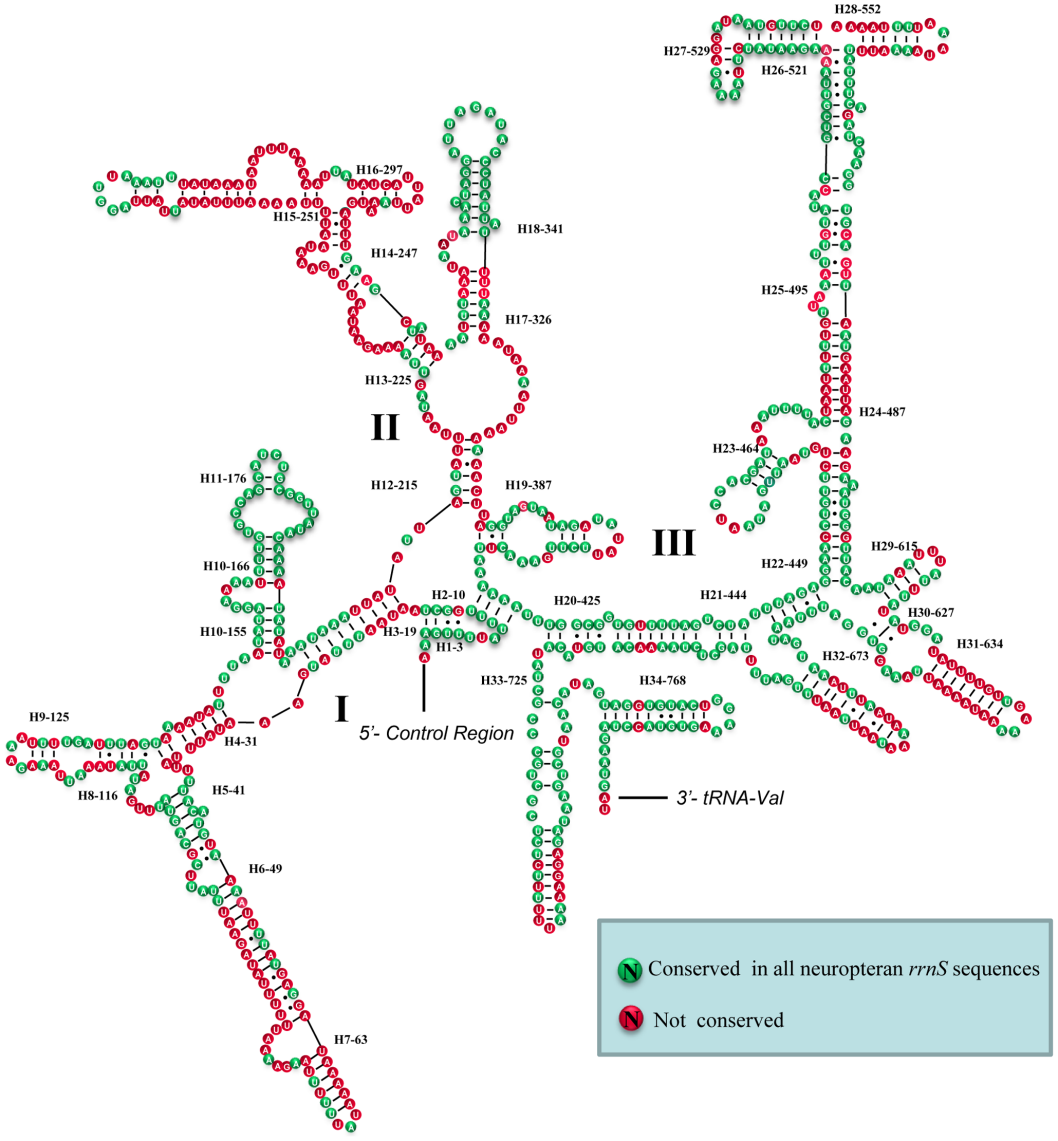
Secondary structure of *T. langii rrnS*. Each helix is numbered progressively from the 5′ to 3′ end together with the first nucleotide belonging the helix itself. Domains are labeled with Roman numbers. Dashes (−) indicate Watson-Crick base pairing and dots (•) indicate unmatched base pairing.

**Figure 4:**
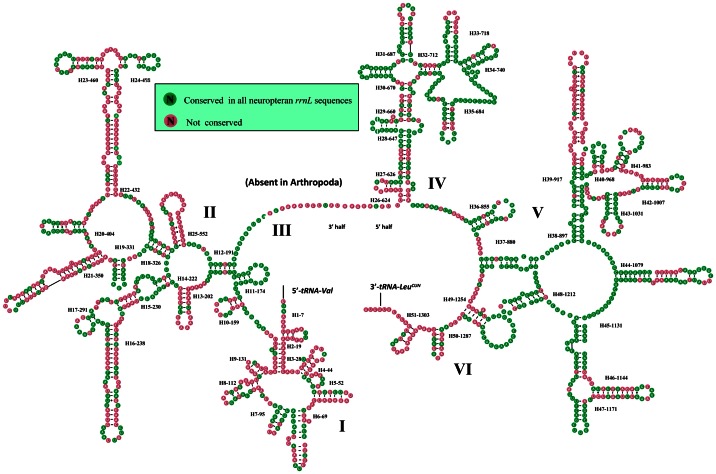
Secondary structure of *T. langii rrnL*. Each helix is numbered progressively from the 5′ to 3′ end together with the first nucleotide belonging the helix itself. Domains are labeled with Roman numbers. Dashes (−) indicate Watson-Crick base pairing and dots (•) indicate unmatched base pairing.

The large and small rRNA subunits (*rrnL* and *rrnS*) in *T. langii* are located at tRNA*^Leu(CUN)^* – tRNA*^Val^* and tRNA*^Val^* – control region, respectively (Fig. 1 and Table S1). The length of *rrnS* and *rrnL* is determined to be 798 bp and 1,325 bp, respectively. The inferred secondary structure models for *rrnS* and *rrnL* are provided in Fig. 3 and Fig. 4, respectively. The secondary structure of both *rrnS* and *rrnL* are largely in agreement with the secondary structure models proposed for other endopterygote orders [29]–[31]. The secondary structure of *T. langii rrnL* consists of six structural domains (domain III is absent in arthropods) and 51 helices (Fig. 4), and the *rrnS* is comprised of three structural domains and 34 helices (Fig. 3). As domain I in *rrnL* is highly variable among insects and difficult to predict, most previous studies have no proposed models for this region [29]–[31]. Negrisolo et al. provided the first predicted secondary structure of domain I for *rrnL* of *Libelloides macaronius* [18]. Compared with this model, nine helices in domain I of *T. langii rrnL* were predicted rather than eight helices in *L. macaronius*.

The multiple alignment of neuropteran *rrnS*s spanned 813 positions and contained 319 variable sites including 168 parsimony-informative sites. Nucleotide variability among domains and helices was unevenly distributed. The helices (H11-176, H18-341) exhibit almost 100%INUC, while some others (i.e. H6-49, H7-63, H31-634) display very low %INUC. The multiple alignment of neuropteran *rrnL*s extended over 1376 positions and contained 583 variable sites, including 321 parsimony-informative sites. Conserved nucleotides were unevenly distributed throughout the *rrnL* secondary structure, with the highest level of non-varying positions located on domains IV–V and lowest levels observed in domains I–II (Fig. 4).

### Protein-coding genes

The mt genome of *T. langii* includes the canonical 13 PCGs that are also present in all other sequenced lacewings and most animal mt genomes [15], [18], [19]. The orientation of PCGs is identical in the mt genomes of all sequenced lacewings: nine in J-strand and four in N-strand. All but one PCG of *T. langii* initiate with ATN as the start codon (seven with ATG, four with ATT and one with ATC) (Table S1). The only exception is the *COI* gene, which uses non-canonical TCG as a start codon. The unusual start condons for the *COI* gene in Neuroptera is a common feature and include ACG for *L. macaronius* [18] and *Myrmeleon immaculatus* [15]; TTA for *Ascaloptynx appendiculatus*; TCG for *Polystoechotes punctatus* [17], *Chrysoperla nipponensis* and *Apochrysa matsumurae* [19] respectively. It is a widespread phenomenon in Neuroptera and other endopterygote insects that the *COI* gene often starts with non-standard putative codons [32]–[34].

Nine PCGs of *T. langii* share the complete termination codons TAA (*ND2*, *ATP8*, *ATP6*, *COI*, *COIII*, *ND4L* and *ND6*) or TAG (*ND1* and *ND3*), while the remaining four had incomplete termination codons T (*ND4*, *ND5*, *COII* and *CytB*) (Table S1), which is also common in other lacewings. The truncated stop codons are suggested to be simply a product of the selective pressure to reduce genome size [35], [36] and are presumed to be completed via post-transcriptional polyadenylation [37].

### Nucleotide composition and codon usage

The nucleotide composition of the *T. langii* mt genome was biased toward A and T as in most other insects. The A+T content of the whole genome, PCGs, tRNAs, rRNAs and the control region is 76.7%, 74.3%, 77.4%, 80.4% and 88.5%. The base composition of nucleotide sequences can be described by its skewness: the whole genome and the control region exhibit the negative AT- and GC-skews while the rRNAs show the opposite situation; the PCGs and tRNAs have the negative AT- and positive GC-skews (Table S2).

Analysis of base composition at each codon position of the concatenated 13 PCGs showed that the third codon position (88.9%) was higher in A+T content than the first (68.0%) and second (66.2%) codon positions (Table S2). Nucleotide frequencies in all codon positions differ between the two strands. In the J-strand, the frequencies of nucleotides are T>A>G>C at the first codon position, T>C>A>G at the second codon position and T>A>C>G at the third codon position. In the N-strand, all three codon positions show the common feature (T>A>G>C).

**Figure 5:**
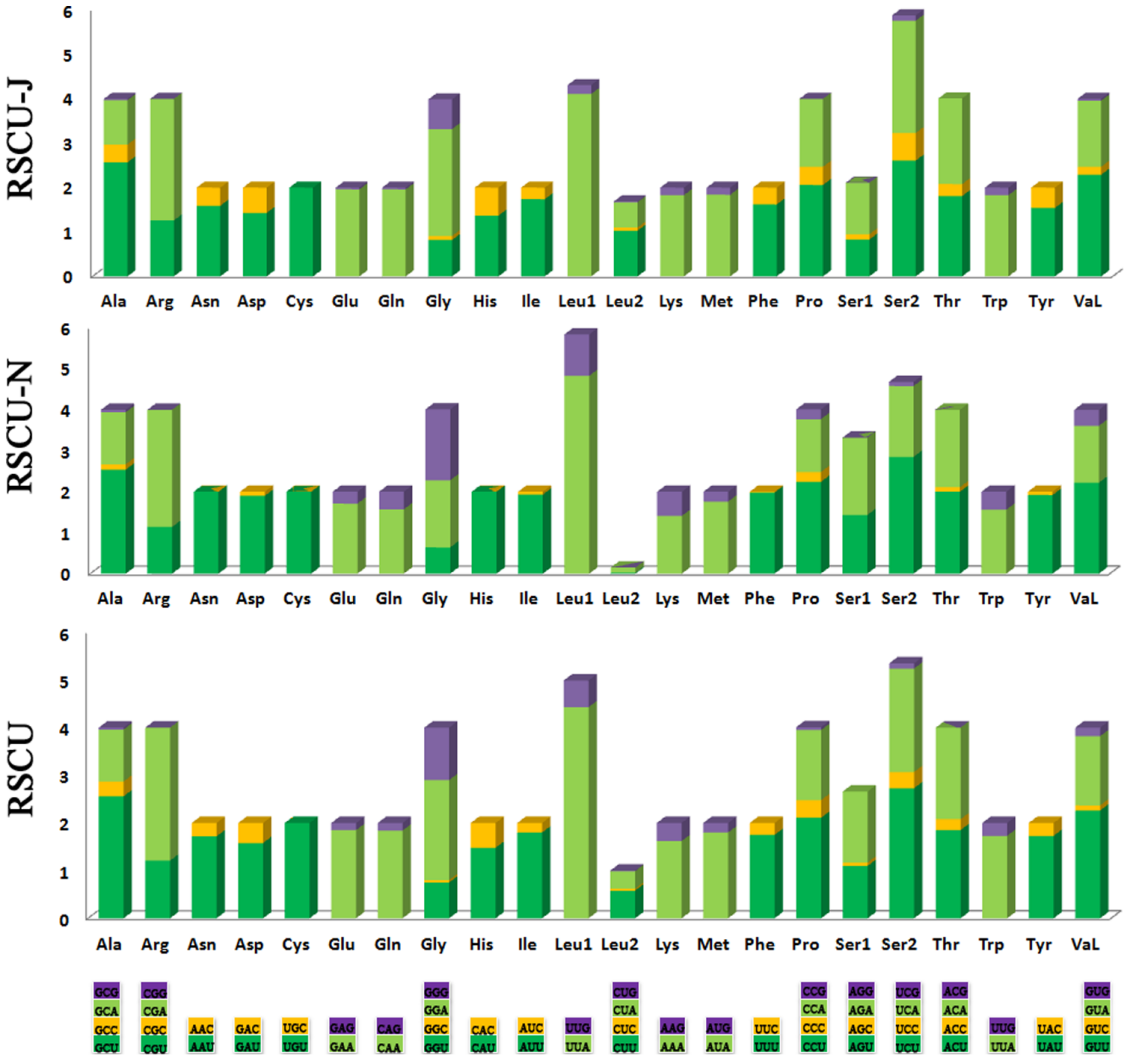
Relative synonymous codon usage (RSCU) in the (N) and (J) strands of the *T. langii* mt genome. Codon families are provided on the x-axis. Along with the different combinations of synonymous codons that code for that amino acid.

The nucleotide bias was also reflected in the codon usage. Four most frequently used codons, TTA (leucine), ATT (isoleucine), TTT (phenylalanine) and ATA (methionine), were all composed wholly of A and/or T. The two-fold degenerate codon usage also demonstrated bias favoring T/A over C/G at the third codon position on both stands. The four-fold degenerate codon usage presented a definite bias towards thymine at the third codon position of PCGs except Gly (GGN), Arg (CGN) and Ser (AGN), whose most frequently used codons terminated with adenine (Fig. 5).

### Non-coding region

The longest non-coding region (1,358 bp; putative control region) is flanked by *rrnS* and *tRNA^Ile^* with 88.5% A+T content, and is the most AT-rich region in the mt genome of *T. langii*. Unlike hymenopteran [38] or hemipteran [34] insects, this region in the mt genome of sequenced lacewings is comparatively simple, with little evidence of conserved blocks or long tandem repeats. The abundance of A/T is due to the repeated motifs containing mostly/only A/T of different length. The most abundant AT motif occurs 231 times, AAT occurs 91 times, AATT occurs 62 times, AATTT occurs 24 times, AATTTTT occurs 12 times, and TTATATAT occurs six times. The motifs are represented by shorter identical strings as well as longer consensus patterns sharing 80% of identical positions. When an 80% minimum identity was allowed among repeated motifs, two stings were identified in the control region of *T. langii*, each containing 41 nucleotides, respectively, from base 224 to base 264 and from 428 to 468. Their consensus sequence was ATTT(T/A)AAA(GA/AG)AATAAATTTAAAA(T/C)CGCAGTTTCTCCCTAAA. This trend also occurs in other neuropteran insects [18].

In addition, some short tandem repeat sequences were detected in the control region of *T. langii*, such as (ATTTT)_4_, (TAATATAA)_2_, (AT)_4_, (AT)_6_, (AT)_7_, and (AT)_8_. These tandem repeat elements can be considered as microsatellites and are useful in the study of geographical structure and phylogenetic relationship of species [25]. Thus, the utility of the AT-rich region as a phylogenetic marker should be most effective at low taxonomic level (family level and below).

Outside the control region, there are 128 bp non-coding sequences in 13 intergenic regions of *T. langii*, ranging in size from one to 63 bp (Table S1). Three of them spanned more than 15 bp. The longest spacer sequence (63 bp) exists between *tRNA^Ile^* and *tRNA^Gln^* which is also present in all part/fully sequenced neuropteran mt genomes, varying from 33 bp to 66 bp; this spacer diverged relatively very fast among different families of the lacewings [18]. The third longest insertion of *T. langii* is located between *ND1* and *tRNA^Ser(UCN)^*, which shares the conserved motif (TTAAATNNNNTNAGAT, N = A/T) across Neuroptera. The conservation of this region is also known in many other insects, which is shown to be a binding site for a bidirectional transcription termination factor (DmTTF) [39].

### The assessment of base compositional heterogeneity

The total AT% of all included neuropteran species ranged between 74.50% and 79.10% with a mean of 77.63%. The total AT% of megalopteran species ranged between 74.90% and 78.30% with a mean of 76.33%. The AT% of the species from Raphidioptera was 80.30%.

The assessment of base-compositional heterogeneity in the neuropteridan dataset shows that the overall dataset exhibited the complex pattern of compositional bias. To assess the base compositional heterogeneity, we made 55 pair wise comparisons to calculate the disparity index (*I*_D_). When we compared all 13 PCGs simultaneously, 48 comparisons had a statistically significant heterogeneous substitution pattern. In general, the *I*_D_ was low between the taxa with lower ranges of AT% and very high between the taxa with wider ranges of AT%. Eight of eleven neuropteridan species exhibited a similar base composition with a mean *I*_D_ between four and six in the dataset (PCG123), suggesting the overall dataset exhibited a high level of base compositional heterogeneity (Table 1). The dataset (PCG12) (removal of fast evolving sites) shows a lower level of base compositional heterogeneity than the dataset (PCG123) with a mean *I*_D_ between one and two in most taxa. The *I*_D_ values calculated from individual codon partitions suggested that the level of base compositional heterogeneity was the lowest in PCG2, followed by PCG1 and PCG3 (Table 1). Among nucleotide sequences, the ranked order of the datasets from best to worst was: PCG2 > PCG1 > PCG12 > PCG3 > PCG123.

**Table 1:** Disparity index values calculated from pairwise comparisons among neuropteridan species included in this study.

Taxa	PCG123		PCG12		PCG1		PCG2		PCG3	
	Sum	Mean	Sum	Mean	Sum	Mean	Sum	Mean	Sum	Mean
*Ascaloptynx*	50.08	5.01	24.60	2.46	24.85	2.49	6.26	0.63	35.90	3.60
*Apochrysa*	50.76	5.01	15.05	1.51	18.63	1.86	3.58	0.36	57.43	5.74
*Chrysoperla*	56.15	5.61	15.27	1.53	24.62	2.46	3.67	0.37	75.05	7.51
*Corydalus*	62.00	6.20	22.43	2.24	28.27	2.83	6.79	0.68	86.54	8.65
*Ditaxis*	43.72	4.37	17.51	1.75	19.15	1.92	4.27	0.43	37.73	3.77
*Libelloides*	95.45	9.55	47.43	4.74	35.10	3.51	3.98	0.40	103.37	10.34
*Mongoloraphidia*	65.74	6.47	31.98	3.20	50.17	5.02	26.41	2.61	29.24	2.92
*Polystoechotes*	56.27	5.62	12.97	1.30	20.78	2.08	4.46	0.45	91.73	9.17
*Protohermes*	41.42	4.14	15.96	1.60	18.23	1.82	6.93	0.93	38.64	3.86
*Thyridosmylus*	36.48	3.65	13.30	1.33	18.34	1.83	4.39	0.44	35.07	3.51
*Sialis*	27.66	2.77	11.42	1.14	12.38	1.24	3.37	0.34	26.51	2.65

‘Sum’ indicates the sum of all *I*_D_ calculated for a particular taxon, and ‘Mean’ indicates the average of *I*_D_ for that taxon. Taxa with high *I*_D_-values have a significantly different base compositional bias from the rest of the species. Numeric values shown next to PCG (i.e. PCG1) represent codon positions. The values shown here highlight the level of base compositional heterogeneity in our dataset.

### Phylogenetic analyses

Base-compositional heterogeneity and branch length heterogeneity are known to be major issues with insect mt genomes in general and neuropteridan/coleopteran mt genomes in particular [8], [40]. According to the test of base-compositional heterogeneity in the neuropteridan dataset herein, and previous studies [40], [41], the datasets of PCG12 and AA are less likely to violate the ‘stationarity’ assumptions, which imply that base composition is constant over all lineages in the dataset [42]. To alleviate the influence of base-compositional heterogeneity and any concerns of possible long-branch attraction, we used a range of phylogenetic approaches to recover the neuropteridan relationships and optimized outgroup selection empirically based on short branches with *Trachypachus holmbergi* (Coleoptera), *Adoxophyes honmai* (Lepidoptera) and *Drosophila melanogaster* (Diptera).

**Figure 6:**
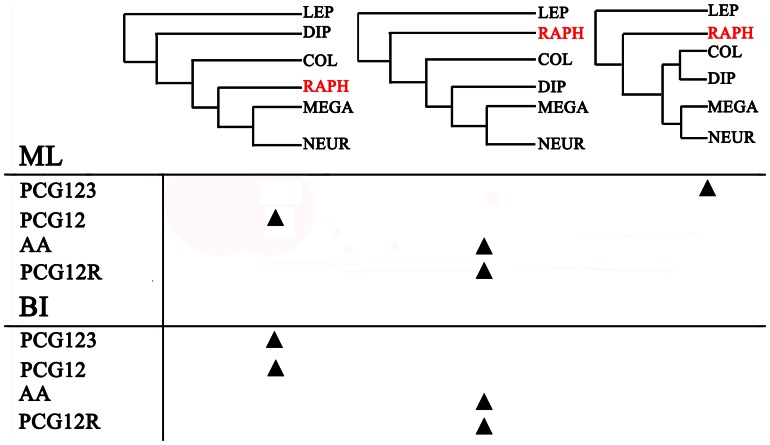
Interordinal relationships inferred by different datasets and analytical methods. The triangle indicates that the most likely tree recovered for a particular dataset/analysis combination supports one of the three topologies listed. COL: Coleoptera, DIP: Diptera; LEP: Lepidoptera; MEGA: Megaloptera; NEUR: Neuroptera; RAPH: Raphidioptera.

**Figure 7:**
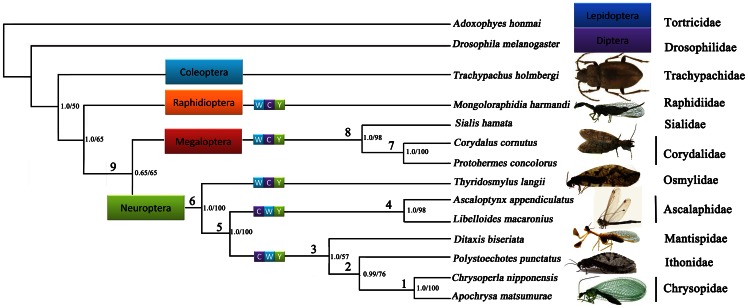
Phylogenetic tree inferred from the sequenced Neuropterida mt genomes. Phylogenetic analyses are based on the PCG12 dataset. Numbers close to the branching points are ML bootstrap support values (right) and Bayesian posterior probabilities in percentages (left). Numbers above each node relate to values provided in Table 2 for different support statistics for that node recovered by different data combinations.

Phylogenetic analyses of the four datasets yielded three different patterns of the relationships between the holometabolan orders (Fig. 6). Examination of the trees recovered from these analyses indicated that each analysis produced identical topologies (Fig. 7), with respect to neuropteran and megalopteran relationships. Bayesian posterior probabilities and bootstrap values of ML of major nodes recovered by various phylogenetic approaches are listed in Table 2. Within Neuropterida, a sister relationship between Megaloptera and Neuroptera was recovered in all analyses of different datasets (node  = 9) with high support, which is consistent with the results from the previous mt genome phylogeny of Neuropterida [15], [16]. The largest difference is the uncertain position of Raphidioptera among the different datasets. The datasets of PCG12 (BI and ML) and PCG123 (BI) recovered identical tree topologies which supports a monophyletic Neuropterida: ((Neuroptera + Megaloptera) + Raphidioptera). The datasets (PCG12R and AA) of both BI and ML analyses fail to support a monophyletic Neuropterida as Neuroptera + Megaloptera are recovered as the unlikely sister group to Diptera.

**Table 2:** Bayesian posterior probabilities and bootstrap values of ML recovered by various phylogenetic approaches.

Relationship	PCG123		PCG12		AA		PCG12R	
	ML	BI	ML	BI	ML	BI	ML	BI
1	100	1.0	100	1.0	100	1.0	100	1.0
2	63	0.98	76	0.99	84	1.0	64	0.99
3	94	1.0	57	1.0	95	1.0	72	1.0
4	100	1.0	98	1.0	100	1.0	100	1.0
5	100	1.0	100	1.0	100	1.0	80	1.0
6	100	1.0	100	1.0	100	1.0	100	1.0
7	100	1.0	100	1.0	100	1.0	100	1.0
8	100	1.0	98	1.0	97	1.0	100	1.0
9	86	1.0	65	0.65	100	1.0	97	1.0

Relationships 1–9 refer to the nodes 1–9 in Figure 7.

Among different datasets, PCG12 that exhibits a lower level of base compositional heterogeneity seems most effective at solving the order level relationships. The uncertain position of Raphidioptera that differs in the phylogenetic trees from different datasets may be also influenced by branch length heterogeneity. There exists some variation in the branch lengths within the taxa (Table S3). The families from Megaloptera and Neuroptera show similar uniform branch lengths. The single exemplar of Raphidioptera shows significantly longer branches than that for Megaloptera and Neuroptera. Based on the limited sample of Raphidioptera and the influence of branch length heterogeneity, the position of Raphidioptera is still uncertain in this study. Increased taxon sampling within Raphidioptera, from both families Inocellidae and Raphidiidae, will likely help solve this problem.

The monophyly of Neuroptera is an undoubted hypothesis strongly supported by both larval features [20], [43] and molecular data [11] that is also demonstrated in our study based on different datasets and methods. Comparative analysis of 11 complete mt genomes sequenced to date reveals some important and novel insights into the phylogeny and evolution of mt gene organization of Neuropterida. All nodes among Neuroptera were perfectly supported in both analyses except that subtending Mantispidae and Ithonidae. Due to the limited number of mt genomes sequenced, the family–level relationships within Neuroptera remain still largely unresolved in our analyses, but important patterns are evident. Our data does not support the proposed division of Neuroptera into three suborders (i.e. Nevrorthiformia, Myrmeleontiformia and Hemerobiiformia) by Aspöck et al. (2001) and reflects the results of most subsequent authors where only Myrmeleontiformia are only recovered as monophyletic, with Hemerobiiformia being paraphyletic relative to both Myrmeleontiformia and Nevrorthiformia [8], [12], [13], [44]. The ancestral gene arrangement in Osmylidae presented here represents important additional evidence as to the paraphyly of Hemerobiiformia. In morphological analyses based on characteristics of adults and larvae and on larval and adult head structures [43], [45], Osmylidae were placed in a weakly supported clade Hemerobiiformia. This is contrary to all analyses using molecular data separately [12], [44] or in combination with morphological data [8] that consistently recover Osmylidae, along with Sisyridae and Nevrorthidae near the base of the phylogeny, clearly reinforcing the apparent antiquity of the origin of this family. Osmylidae, together with Nevrorthidae and Sisyridae, also share an apparent synapomorphy of enlarged female external gonocoxites 9 [13] and have a series of sympleisiomorphies characters indicative of the likely early cladogenesis of these families during the Late Permian or Early Triassic [8]. The basal position of Osmylidae within Neuroptera is confirmed in our study with high support nodes recovered by different datasets (node  = 6).

As apparently one of the oldest and most primitive groups of Neuroptera [13], Osmylidae also exhibit the plesiomorphic condition of gene order found throughout the rest of Neuropteroidea (i.e. WCY) (Fig. 7). On the basis of the irreversible process of gene rearrangement and using estimates of divergence times by Winterton et al. (2010) [8], then the translocation to the derived gene order in higher Neuroptera may be occurred somewhere around 260 million years ago, after the divergence of the Osmylidae ancestor. Further work is needed to confirm with the more sequenced mitochondrial genomes in Neuroptera to determine the timing of this gene rearrangement more precisely.

## Conclusions

This work has added to our current knowledge of gene arrangement of Neuroptera. The first complete mt genome of *T. langii* (Neuroptera: Osmylidae) presented here displays an ancestral gene organization that lacks translocation of *trnC* as shared by all previously sequenced neuropteran mtDNAs. This discovery reveals that the *trnC* translocation is not synapomorphic for the entire neuropteran clade but only for higher Neuroptera. Comparative analyses suggest that gene junctions, tRNAs and rRNAs are comparatively conserved among the neuropteran mt genomes. The mt genomic phylogeny of different datasets and methods herein reconstructed clearly supports the monophyly of Neuroptera, the more basal position of Osmylidae and the sister relationship between Megaloptera and Neuroptera. However, based on the limited taxon sampling and the influence of base compositional and branch length heterogeneity, only the dataset of PCG12 in BI and ML analyses and PCG123 in BI analyses recovered identical tree topologies, which support a monophyletic Neuropterida: ((Neuroptera + Megaloptera) + Raphidioptera). To more fully elucidate phylogenetic relationships within Neuropterida based on comparative analysis of mt genomes, further study is needed based on increased taxon sampling within major groups of Neuropterida, especially representatives of neuropteran families such as Coniopterygidae, Dilaridae, Hemerobiidae, Nevrorthidae and Sisyridae, and well as Inocellidae (Raphidioptera).

## Materials and Methods

### Sample origin and genomic DNA extraction

Specimens of *Thyridosmylus langii* were collected from Motuo, Tibet Autonomous Region, China in August 2011. All specimens were preserved in 100% ethanol and stored at −20°C until DNA extraction. Total genomic DNA was extracted from thorax muscle tissue and the leg muscle tissue using the TIANamp Genomic DNA Kit in accordance with the manufacturer's instructions. Voucher specimens (Nos. NEU-0018), preserved in alcohol, are deposited at the Entomological Museum of China Agricultural University (Beijing).

### PCR amplification, cloning and sequencing

PCR amplification was performed using a set of insect universal primers [46], [47] and primers specifically designed on the *Thyridosmylus langii* sequences (Table S4). Initially, 13 fragments were amplified using the universal primers from previous work (Fig. 1). On the basis of these short fragments for secondary PCRs, Seven perfectly matching primers were designed by using Primer Premier 5.0 to bridge the gaps.

Short PCRs were conducted using Qiagen Taq DNA polymerase (Qiagen, Beijing, China) with the following cycling conditions: 5 min at 94°C, followed by 35 cycles of 50 s at 94°C, 30 s at 40–55°C, and 1–2 min at 72°C. The final elongation step was continued for 5 min at 72°C. Long PCRs were performed using NEB Long Taq DNA polymerase (New England Biolabs) under the following cycling conditions: 30 s at 95°C, followed by 45 cycles of 10 s at 95°C, 50 s at 48–55°C, and 3–5 min at 65°C. The final elongation was continued for 10 min at 65°C. These PCR products were detected via electrophoresis in a 1% agarose gel and stained with ethidium bromide.

All PCR Products were sequenced in both forward and reverse directions using the BigDye Terminator Sequencing Kit (Applied Bio Systems) and the ABI 3730XL Genetic Analyzer (PE Applied Biosystems, San Francisco, CA, U.S.A.) with two vector-specific primers and internal primers for primer walking.

### Sequence analysis and inferences of secondary structures

Original sequence files were manually checked and assembled using the software BioEdit 7.0.5.3 [48], Chromas 1.62 and DNAMAN 6.0. The tRNAs were identified by tRNAscan-SE Search Server v.1.21 [27] with default setting or recognized manually by comparison with published neuropteran mt sequences (i.e. *Polystoechotes punctatus* (Ithonidae), *Libelloides macaronius* (Ascalaphidae) and *Chrysoperla nipponensis* (Chrysopidae)) [15], [18], [19]. Secondary structures of the small and large ribosomal RNAs were inferred using alignment to the models predicted for *L. macaronius*, which is largely in agreement with those proposed for other endopterygote orders (i.e. Coleoptera, Diptera, Hymenoptera, and Lepidoptera) [4], [18], [30], [49]. PCGs were identified using Clustal X [50] by similarity of published neuropteran mt gene amino acid sequences. Translation of PCG open reading frames were further analyzed by MEGA version 4.0 [51] for the codon usage under the condition of the invertebrate mtDNA genetic code. Sequence motifs in the AT-rich region were identified using the Spectral Repeat Finder program [52]. Strand asymmetry was calculated using the formulae: AT skew =  [A−T]/[A+T] and GC skew =  [G−C]/[G+C] for the strand encoding the majority of the PCGs [53].

### Assessment of the degree of heterogeneity

To measure the variation in evolutionary pattern, we calculated the disparity index (*I*_D_) [54] for all 13 PCGs together and pairwise. We also tested the homogeneity of substitution pattern (*I*_D_ test) using a Monte–Carlo method with 1000 replicates as implemented in MEGA 4.0 [51]. We calculated the probability of rejecting the null hypothesis that sequences have evolved with the same pattern of substitution at α<0.01. All positions containing gaps and missing data were removed from the data set (complete deletion option). Also to assess the degree of branch length heterogeneity among Neuropterida, we extracted branch length estimates from the most likely tree, after Maximum-likelihood and Bayesian analysis of the datasets. Branch lengths were conveniently displayed on this tree, using FigTree (v. 1). We then manually calculated the branch length for each taxon, from the tip to the basal node of the tree.

### Phylogenetic analysis

Phylogenetic analysis was carried out based on the 13 complete mt genomes of neuropteridan and endopterygote outgroups from Genbank (Table S5) in addition to the one presented here for Osmylidae. Within Endopterygota, the closest living relatives of Neuropterida are the beetles [11], [15], so a representative of Coleoptera and two species from Diptera and Lepidoptera respectively were selected for outgroup comparison. All PCGs were aligned at the amino acid level using the default settings in Mega version 4.0 [51]. The alignments were back-translated into the corresponding nucleotide sequences. The stop codons of PCGs were excluded when aligned. Two rRNAs were respectively aligned in Mega version 4.0 [51] as nucleotides. Ambiguously aligned regions of PCGs and rRNAs were checked by eye.

To overcome the base compositional heterogeneity and branch length heterogeneity and explore the effect of method choice on recovery of monophyly of Neuropterida, four datasets were used for phylogenetic analyses: 1) a concatenated nucleotide sequence alignment of PCGs (PCG123); 2) a concatenated nucleotide sequence alignment of the first and the second codon positions of PCGs (PCG12); 3) a concatenated nucleotide sequence alignment of the first and the second codon positions of PCGs and two rRNA genes (PCG12R); and 4) a concatenated amino acid sequence alignment of PCGs (AA). We analyzed these datasets in both Bayesian inference (BI) and Maximum-likelihood (ML) frameworks.

Partitioned ML and Bayesian analyses were run with PCG123, PCG12, PCG12R, and AA matrix, using PHYML online web server [55] and MrBayes Version 3.1.1 [56]. The best-fit model for the amino acid sequence alignment was determined with ProtTest [57] and the jModelTest 0.1.1 [58] was used for the nucleotide sequence of each gene, according to the Akaike Information Criterion (AIC). GTR+I+G model for nucleotide sequence and MtREV model for amino acid sequence were used to optimize the topology. For Bayesian analyses, two simultaneous runs of 5 to 10 million generations were conducted for the matrix and trees were sampled every 1,000 generations, with the first 25% discarded as burn-in. Stationarity was considered to be reached when the average standard deviation of split frequencies was below 0.01. In ML analysis, the parameters were estimated during analysis and the node support values were assessed by bootstrap resampling (BP) [59] calculated with 1000 replicates.

## Supporting Information

Table S1 **Organization of** ***Thyridosmylus langii*** **mt genome.**
(DOCX)

Table S2 **Nucleotide composition of the** ***Thyridosmylus langii*** **mt genome.**
(DOC)

Table S3 **Comparision of branch lengths among Neuropterida insects.**
(DOCX)

Table S4 **Primer sequences used in this study.**
(DOC)

Table S5 **Mt genomes used in comparative analyses.**
(DOCX)
