# Supplementary material for: Ancestral Gene Organization in the Mitochondrial Genome of Thyridosmylus langii (McLachlan, 1870) (Neuroptera: Osmylidae) and Implications for Lacewing Evolution
Source: PLoS One. 2013 May 23;8(5):e62943. doi: 10.1371/journal.pone.0062943 (PMC3662673; doi:10.1371/journal.pone.0062943)
Supplement: Table S1 — Organization of Thyridosmylus langii mt genome. (DOCX) [file pone.0062943.s001.docx]

**Table S1 Organization of *Thyridosmylus langii* mt genome.**

| **Gene** | **Direction** | **Location (bp)** | **Size (bp)** | **Anticodon** | **Start Codon** | **Stop Codon** | **Intergenic**  **Nucleotide^*^** |
| --- | --- | --- | --- | --- | --- | --- | --- |
| *tRNA^Ile^* | F | 1-67 | 67 | 32-34 GAT |  |  | 0 |
| *tRNA^Gln^* | R | 131-199 | 69 | 167-169 TTG |  |  | 63 |
| *tRNA^Met^* | F | 201-273 | 73 | 231-233 CAT |  |  | 1 |
| *ND2* | F | 274-1290 | 1017 |  | ATG | TAA | 0 |
| *tRNA^Trp^* | F | 1289-1356 | 68 | 1319-1321 TCA |  |  | -2 |
| *tRNA^Cys^* | R | 1349-1414 | 66 | 1383-1385 GCA |  |  | -8 |
| *tRNA^Tyr^* | R | 1415-1480 | 66 | 1447-1449 GTA |  |  | 0 |
| *COI* | F | 1482-3020 | 1537 |  | TCG | TAA | 1 |
| *tRNA^Leu(UUR)^* | F | 3029-3093 | 65 | 3058-3060 TAA |  |  | 10 |
| *COII* | F | 3095-3779 | 685 |  | ATG | T- | 1 |
| *tRNA^Lys^* | F | 3780-3850 | 71 | 3810-3812 CTT |  |  | 0 |
| *tRNA^Asp^* | F | 3853-3916 | 64 | 3882-3884 GTC |  |  | 2 |
| *ATP8* | F | 3917-4075 | 159 |  | ATT | TAA | 0 |
| *ATP6* | F | 4069-4746 | 678 |  | ATG | TAA | -7 |
| *COIII* | F | 4746-5534 | 789 |  | ATG | TAA | -1 |
| *tRNA^Gly^* | F | 5537-5602 | 66 | 5567-5569 TCC |  |  | 2 |
| *ND3* | F | 5603-5956 | 354 |  | ATT | TAG | 0 |
| *tRNA^Ala^* | F | 5955-6020 | 66 | 5984-5986TGC |  |  | -2 |
| *tRNA^Arg^* | F | 6020-6083 | 64 | 6048-6050 TCG |  |  | -1 |
| *tRNA^Asn^* | F | 6084-6149 | 66 | 6115-6117 GTT |  |  | 0 |
| *tRNA^Ser(AGN)^* | F | 6152-6220 | 69 | 6179-6181 GCT |  |  | 2 |
| *tRNA^Glu^* | F | 6220-6284 | 65 | 6250-6252 TTC |  |  | -1 |
| *tRNA^Phe^* | R | 6283-6346 | 64 | 6314-6316 GAA |  |  | -2 |
| *ND5* | R | 6344-8078 | 1735 |  | ATC | T | -3 |
| *tRNA^His^* | R | 8076-8141 | 66 | 8109-8111 GTG |  |  | -3 |
| *ND4* | R | 8142-9477 | 1336 |  | ATG | T- | 0 |
| *ND4L* | R | 9470-9767 | 298 |  | ATG | TAA | -8 |
| *tRNA^Thr^* | F | 9770-9835 | 66 | 9800-9802 TGT |  |  | 2 |
| *tRNA^Pro^* | R | 9836-9901 | 66 | 9869-9871 TGG |  |  | 0 |
| *ND6* | F | 9904-10425 | 522 |  | ATT | TAA | 2 |
| *CytB* | F | 10431-11562 | 1132 |  | ATG | T | 5 |
| *tRNA^Ser(UCN)^* | F | 11563-11629 | 67 | 11592-11594 TGA |  |  | 0 |
| *ND1* | R | 11647-12576 | 930 |  | ATT | TAG | 17 |
| *tRNA^Leu(CUN)^* | R | 12599-12662 | 64 | 12631-12633 TAG |  |  | 22 |
| *lrRNA* | R | 12663-13987 | 1325 |  |  |  | 0 |
| *tRNA^Val^* | R | 13988-14058 | 71 | 14023-14025 TAC |  |  | 0 |
| *srRNA* | R | 14059-14856 | 798 |  |  |  | 0 |
| Control region |  | 14857-16221 | 1358 |  |  |  | 0 |

“*”: Negative numbers indicate that adjacent genes overlap.
