# Supplementary material for: Ancestral Gene Organization in the Mitochondrial Genome of Thyridosmylus langii (McLachlan, 1870) (Neuroptera: Osmylidae) and Implications for Lacewing Evolution
Source: PLoS One. 2013 May 23;8(5):e62943. doi: 10.1371/journal.pone.0062943 (PMC3662673; doi:10.1371/journal.pone.0062943)
Supplement: Table S2 — Nucleotide composition of the Thyridosmylus langii mt genome. (DOC) [file pone.0062943.s002.doc]

**Table S2 Nucleotide composition of the *Thyridosmylus langii* mt genome.**

|  | **Proportion of nucleotides No. of** | | | | | | | |
| --- | --- | --- | --- | --- | --- | --- | --- | --- |
| **Feature** | **%T** | **%C** | **%A** | **%G** | **%A+T** | **AT Skew** | **GC Skew** | **nucleotides** |
| **Whole genome** | 38.9 | 14.1 | 37.8 | 9.2 | 76.7 | -0.01 | -0.21 | 16221 |
| **Protein-coding genes** | 43.3 | 12.5 | 31.0 | 13.1 | 74.3 | -0.17 | 0.02 | 11142 |
| First codon position | 37.0 | 11.9 | 31.0 | 19.7 | 68.0 | -0.09 | 0.25 | 3714 |
| Second codon position | 46 | 19.4 | 20.2 | 14.4 | 66.2 | -0.39 | -0.15 | 3714 |
| Third codon position | 47 | 6.1 | 41.9 | 5.4 | 88.9 | -0.06 | -0.06 | 3714 |
| **Protein-coding genes-J** | 41.8 | 15.4 | 31.0 | 11.9 | 72.8 | -0.15 | -0.13 | 6852 |
| First codon position | 34.0 | 15.1 | 31.4 | 19.8 | 65.4 | -0.04 | 0.13 | 2284 |
| Second codon position | 45.0 | 21.6 | 20.7 | 13.1 | 65.7 | -0.37 | -0.25 | 2284 |
| Third codon position | 47.0 | 9.4 | 40.9 | 2.6 | 87.9 | -0.07 | -0.57 | 2284 |
| **Protein-coding genes-N** | 45.8 | 7.9 | 31.1 | 15.2 | 76.9 | -0.19 | 0.32 | 4290 |
| First codon position | 43.0 | 6.9 | 30.3 | 19.4 | 73.3 | -0.17 | 0.48 | 1430 |
| Second codon position | 48.0 | 15.9 | 19.5 | 16.4 | 67.5 | -0.42 | 0.02 | 1430 |
| Third codon position | 46.0 | 1.0 | 43.5 | 9.8 | 89.5 | -0.03 | 0.81 | 1430 |
| **tRNA genes** | 39.3 | 10.1 | 38.1 | 12.5 | 77.4 | -0.02 | 0.11 | 1469 |
| **rRNA genes** | 39.4 | 6.7 | 41.0 | 12.9 | 80.4 | 0.02 | 0.32 | 2132 |
| **Control region** | 45.6 | 7.9 | 42.9 | 3.6 | 88.5 | -0.03 | -0.37 | 1358 |
