# Supplementary material for: Ancestral Gene Organization in the Mitochondrial Genome of Thyridosmylus langii (McLachlan, 1870) (Neuroptera: Osmylidae) and Implications for Lacewing Evolution
Source: PLoS One. 2013 May 23;8(5):e62943. doi: 10.1371/journal.pone.0062943 (PMC3662673; doi:10.1371/journal.pone.0062943)
Supplement: Table S3 — Comparision of branch lengths among Neuropterida insects. (DOCX) [file pone.0062943.s003.docx]

|  | Apoc. | Chry. | Poly. | Dita. | Libe. | Asca. | Thyr. | Prot. | Cory. | Sial. | Mong. |
| --- | --- | --- | --- | --- | --- | --- | --- | --- | --- | --- | --- |
| PCG123 (BI) | 0.37 | 0.35 | 0.32 | 0.36 | 0.42 | 0.41 | 0.35 | 0.38 | 0.40 | 0.31 | 0.64 |
| PCG123 (ML) | 0.42 | 0.36 | 0.35 | 0.42 | 0.52 | 0.51 | 0.42 | 0.48 | 0.52 | 0.48 | 0.70 |
| PCG12 (BI) | 0.23 | 0.23 | 0.20 | 0.20 | 0.25 | 0.24 | 0.20 | 0.23 | 0.24 | 0.18 | 0.45 |
| PCG12 (ML) | 0.31 | 0.31 | 0.30 | 0.32 | 0.34 | 0.33 | 0.28 | 0.31 | 0.33 | 0.25 | 0.60 |

**Table S3 Comparision of branch lengths among Neuropterida insects.**

The first four letters of the species name represents that species. (i.e. Apoc.: *Apochrysa matsumurae* )
