# Supplementary material for: Ancestral Gene Organization in the Mitochondrial Genome of Thyridosmylus langii (McLachlan, 1870) (Neuroptera: Osmylidae) and Implications for Lacewing Evolution
Source: PLoS One. 2013 May 23;8(5):e62943. doi: 10.1371/journal.pone.0062943 (PMC3662673; doi:10.1371/journal.pone.0062943)
Supplement: Table S4 — Primer sequences used in this study. (DOC) [file pone.0062943.s004.doc]

**Table S4 Primer sequences used in this study.**

| No. of fragment | Primer name | Nucleotide sequence (5’-3’) | Reference |
| --- | --- | --- | --- |
| 1 | TM-J210 | AATTAAGCTACTAGGTTCATACCC | Simon et al., 2006 |
|  | C1-N1738 | TTTATTCGAGGGAATGCTATATC | Simon et al., 2006 |
| 2 | N-1476 | TTGGAATCTGAGCAGGCT | Present study |
|  | R-2228 | GTAATCGAATGAAGGAAGAGC | Present study |
| 3 | C1--J2756 | ACATTTTTTCCTCAACATTT | Simon et al., 2006 |
|  | C2--N3665 | CCACAAATTTCTGAACACTG | Simon et al., 2006 |
| 4 | C2-J3399 | TCTATTGGTCATCAATGGTACTG | Simon et al., 2006 |
|  | A8-N4061 | GAAAATAAATTTGTTATCATTTTCA | Simon et al., 2006 |
| 5 | TK-J3790 | CATTAAGTGACTGAAAGCAAGTA | Simon et al., 2006 |
|  | A6-N4552 | ATGACCTGCAATTATATTAGC | Simon et al., 2006 |
| 6 | F3098 | CCGTTTCTCTTTCCTTG | Present study |
|  | R973 | CTTGGTTTCATTCGTGGT | Present study |
| 7 | N3-J5747 | CCATTTGAATGTGGGTTTGACCC | Simon et al., 2006 |
|  | TF-N6384 | TAAATTTAAAGTGTGGTATTGAAG | Simon et al., 2006 |
| 8 | F191 | TACCACGAATGAAA | Present study |
|  | R1039 | CTTCTACTTTAGTGACTGCTGGAG | Present study |
| 9 | N5-J7077 | TTAAATCCTTTGAGTAAAATCC | Simon et al., 2006 |
|  | N5-N7793 | TTAGGTTGAGATGGTTTAGG | Simon et al., 2006 |
| 10 | F415 | TCCAGCAGTCACTAAAGTAG | Present study |
|  | R950 | GAGGGTATGGGTTATTACG | Present study |
| 11 | N4-J8641 | CCAGAAGAACATAGCCCATG | Simon et al., 2006 |
|  | N4L-N9629 | GTTTGTGAAGGTGTGTTGGG | Simon et al., 2006 |
| 12 | N4L-J9648 | TCCCAACACACCTTCACAAAC | Simon et al., 2006 |
|  | N4L-11010 | TATCAACAGCAAATCCTCCTCA | Simon et al., 2006 |
| 13 | CB-J10621 | CTCATACTGATGAAATTTTGGTTC | Simon et al., 2006 |
|  | CB-N11526 | TTCTACTGGTCGTGCTCCAATTCA | Simon et al., 2006 |
| 14 | CB-J11335 | CATATTCAACCAGAATGATA | Simon et al., 2006 |
|  | N1-N12067 | AATCGTTCTCCATTTGATTTTGC | Simon et al., 2006 |
| 15 | N1-J11876 | CGAGGTAAAGTACCACGTACTCA | Simon et al., 2006 |
|  | N1-N12595 | GTTGGATTTCTAACTTTATTRGARCG | Simon et al., 2006 |
| 16 | N1-J12261 | TACCTCATAAGAAATAGTTTGAGC | Simon et al., 2006 |
|  | LR-N13000 | TTACCTTAGGGATAACAGCGTAA | Simon et al., 2006 |
| 17 | LR-J12888 | CCGGTCTGAACTCAGATCATGTA | Simon et al., 2006 |
|  | LR-N13889 | ATTTATTGTACCTTTTGTATCAG | Simon et al., 2006 |
| 18 | LR-J13342 | CCTTAGCACAGTTAAAATACTGC | Simon et al., 2006 |
|  | LR-N14220 | TTATGCACATATCGCCCGTC | Simon et al., 2006 |
| 19 | F435 | TAATAAATCAGTGGGCAGG | Present study |
|  | R14745 | GTGCCAGCATCTGCGGTTATAC | Present study |
| 20 | F-14132 | CAACCTACTATGTTACGACTTATCTC | Present study |
|  | R-1462 | GGAACCTTCATAAATGGG | Present study |
