# Supplementary material for: Ancestral Gene Organization in the Mitochondrial Genome of Thyridosmylus langii (McLachlan, 1870) (Neuroptera: Osmylidae) and Implications for Lacewing Evolution
Source: PLoS One. 2013 May 23;8(5):e62943. doi: 10.1371/journal.pone.0062943 (PMC3662673; doi:10.1371/journal.pone.0062943)
Supplement: Table S5 — Mt genomes used in comparative analyses. (DOCX) [file pone.0062943.s005.docx]

**Table S5 Mt genomes used in comparative analyses.**

| **Order** | **Family** | **Species** | **Accession number** |
| --- | --- | --- | --- |
| **Neuroptera** | Osmylidae | *Thyridosmylus langii* | KC515397 |
|  | Ascalaphidae | *Ascaloptynx appendiculatus* | FJ171324 |
|  |  | *Libelloides macaronius* | FR669150 |
|  | Mantispidae | *Ditaxis biseriata* | FJ859906 |
|  | Ithonidae | *Polystoechotes punctatus* | FJ171325 |
|  | Chrysopidae | *Chrysoperla nipponensis* | AP011623 |
|  |  | *Apochrysa matsumurae* | AP011624 |
| **Megaloptera** | Corydalidae | *Corydalus cornutus* | FJ171323 |
|  |  | *Protohermes concolorus* | EU526394 |
|  | Sialidae | *Sialis hamata* | FJ859905 |
| **Raphidioptera** | Raphidiidae | *Mongoloraphidia harmandi* | FJ859902 |
| **Coleoptera** | Trachypachidae | *Trachypachus holmbergi* | EU877954 |
| **Diptera** | Drosophilidae | *Drosophila melanogaster* | NC_001709.1 |
| **Lepidoptera** | Tortricidae | *Adoxophyes honmai* | NC_008141.1 |
